# Traditional medicine practices for Cutaneous Leishmaniasis in Kalu District, South Wollo, Ethiopia

**DOI:** 10.1186/s41182-025-00804-7

**Published:** 2025-09-30

**Authors:** Massame Tadesse Ergicho, Mulugeta Tamire, Yordanos Tadesse, Stephen L. Walker, Jennifer Palmer, Yohannes Hailemichael, Takele Gezahegn Demie, Tara Mtuy, Endalamaw Gadisa, Mirgissa Kaba, SHARP collaboration

**Affiliations:** 1 School of Public Health, Wolaita Sodo University, Wolaita Sodo, Ethiopia; 2 Department of Preventive Medicine, School of Public Health, College of Health Sciences, Addis Ababa University, Addis Ababa, Ethiopia; 3 Department of Clinical Research, Faculty of Infectious and Tropical Diseases, London School of Hygiene and Tropical Medicine, London, UK; 4 Department of Global Health and Development, Faculty of Public Health and Policy, School of Hygiene and Tropical Medicine, London, UK; 5 Armauer Hansen Research Institute, Addis Ababa, Ethiopia; 6 School of Public Health, St. Paul’s Hospital Millennium Medical College, Addis Ababa, Ethiopia

**Keywords:** Cutaneous leishmaniasis, Traditional treatment, Ethnographic research, Phytomedicine, Plants, Ethiopia

## Abstract

**Background:**

Cutaneous leishmaniasis is a neglected tropical disease of public health importance in Ethiopia, with an estimated 40,000 new cases per year. Access to allopathic diagnostic and treatment facilities is limited. Traditional healthcare is an accessible option in many communities, but there is limited evidence on the types of traditional treatments utilized for cutaneous leishmaniasis in Ethiopia.

**Objective:**

To explore the traditional treatment practices used for cutaneous leishmaniasis in Kalu district, South Wollo, Ethiopia.

**Methods:**

We conducted an ethnographic study from April to August 2023 in Kalu district, Amhara region. Interviews with ten cutaneous leishmaniasis affected individuals, five traditional healers, and three local opinion leaders were conducted to understand their experiences, treatment choices, and perceptions. In addition, observations at three traditional healers were used to document traditional treatment procedures, materials used, and healer-client interactions. The interviews were transcribed verbatim in Amharic and translated to English and thematically analyzed alongside observation notes.

**Results:**

Cutaneous leishmaniasis affected individuals reported using traditional treatments to manage cutaneous leishmaniasis. The factors influencing this choice were lack of awareness about the availability of allopathic treatments, limited access to healthcare facilities, the long duration and high cost of allopathic treatment, trust in traditional healers, and recommendations from community members.

Plant-based remedies were commonly applied to lesions, while other treatments included honey, dried bat meat, application of heated metallic objects, and spiritual practices. Traditional healers recommended various behavioral modifications as part of the therapeutic process to facilitate healing, which included dietary restrictions, limiting farm work and cooking, celibacy, and social isolation.

**Conclusion:**

In Kalu, traditional medicines are the primary source of treatment for cutaneous leishmaniasis. With limited access to allopathic care, cost of services, and trust in local healers, traditional healing of cutaneous leishmaniasis is widely recognized. While further research may help to evaluate the safety and effectiveness of traditional healing practices, there is a need to find ways of engaging healers to support interventions tackling skin diseases.

## Introduction

Leishmaniasis is a neglected tropical disease caused by the protozoa of the genus *Leishmania,* transmitted by the bite of infected female sandflies [1]. Cutaneous leishmaniasis is the most common clinical manifestation of leishmaniasis, with an estimated 700,000 to 1 million new cases worldwide each year [2, 3]. Cutaneous leishmaniasis is endemic in many parts of Ethiopia [4] with over 29 million people at risk and an estimated annual incidence of 40,000 cases [5, 6].

Cutaneous leishmaniasis lesions most commonly occur on exposed parts of the body, particularly the face. Cutaneous leishmaniasis lesions, whether they heal after treatment or spontaneously, typically leave permanent scars which are stigmatizing [7–9], and may lead to exclusion from community activities, and impaired mental health for affected individuals of both sexes [7, 10, 11]. Women with disfiguring scars face additional social consequences, including difficulties in marriage, and some married women experience rejection by their partners due to misconceptions about disease transmission [12].

According to the World Health Organization, traditional medicine plays a role in 65–80% of global healthcare practices [13]. Traditional medicine is the sum total of knowledge, skills, and practices based on the theories, beliefs, and experiences indigenous to different cultures, used to maintain health and treat illness [14]. In Africa, 70–80% of the population relies on it for primary healthcare, and it accounts for 40% of all healthcare services provided [14, 15].

Worldwide, most individuals with cutaneous leishmaniasis self-medicate or seek treatment from traditional healers before visiting a formal healthcare setting [10, 11, 16, 17]. In Ethiopia, diagnostic and treatment facilities for cutaneous leishmaniasis are limited, and most affected individuals rely on traditional treatment options [5, 18]. Among patients who visited health centers, most of them arrived after using traditional treatment for a prolonged period [19]. Cutaneous leishmaniasis affected individuals prefer traditional medicine to allopathic treatment due to lack of trust, high cost of care seeking and treatment, and associated opportunity cost of attending care that requires extended hospitalization [20, 21]. We have previously reported that the catastrophic cost of care seeking is a significant barrier to accessing timely healthcare by individuals affected by cutaneous leishmaniasis in Kalu, Ethiopia [10, 21]. We have also explored the lived experiences of individuals with cutaneous leishmaniasis (referred to as *kunchir* by people in Kalu), which causes significant physical pain and emotional distress. Most participants relied on traditional treatments such as cauterization and plant-based remedies, which often led to burns, scarring, and dissatisfaction [9].

There is a lack of data on the specific traditional practices employed by affected individuals and traditional healers, pathways to seeking treatment, and the reasons for affected individuals’ treatment preferences. We aimed to explore care-seeking pathways and reasons for using traditional medicines, as well as the sources of traditional treatments for cutaneous leishmaniasis in Kalu, Ethiopia.

## Methods and materials

### Study setting

The study was conducted in Kalu district in South Wollo Zone, Amhara Region. Kombolcha, the district capital, is 376 km north-east of Addis Ababa. The district consists of highland, midland, and lowland agroecological settings. Kalu district is an endemic area for cutaneous leishmaniasis, where most affected individuals rely on traditional treatment. However, the nearest cutaneous leishmaniasis diagnosis and treatment service is at Boru Meda General Hospital, 50 km from Kombolcha [10].

This study was conducted as part of the Skin Health Africa Research Programme (SHARP), a multidisciplinary initiative aimed at understanding and improving experiences of severe and stigmatizing skin diseases.

### Study approach and data collection

We conducted a qualitative study using an ethnographic design from April to August 2023. This allowed for direct interaction with traditional healers and other community members, providing a deep understanding of traditional healing practices and community beliefs related to cutaneous leishmaniasis.

The data were collected through in-depth interviews with cutaneous leishmaniasis affected individuals and traditional healers, key informant interviews with local opinion leaders, as well as participatory observations. Semi-structured interview guides, with open-ended and probing questions, were prepared in English and translated to Amharic to guide evidence generation. The questions were developed in line with the research questions and review of relevant literature, and were then refined through consultation with a senior researcher to ensure cultural appropriateness and relevance to the study context.

The interview guide for affected individuals explored care-seeking pathways, treatment experiences, and perceptions of traditional healing for cutaneous leishmaniasis. The traditional healers’ guide helped to document practitioner characteristics, treatment practices, and the sources of traditional medicines for cutaneous leishmaniasis, while the local opinion leader guide captured community-wide perceptions of health and treatment.

All interviews were conducted in Amharic and audio recorded at the participant's home or a location of their choice where they felt comfortable. A quiet place was chosen to avoid distractions and provide privacy.

An observation guide was used for the participatory observation to systematically record the treatment setting, healer-patient interactions, and the traditional treatment processes. Active overt observation was employed, in which the researcher openly observed the participants and their activities. The purpose of the study was clearly explained to the traditional healers’ clients, and oral informed consent was obtained from them before the observation. To reduce the observer effect, trust and rapport were built before the observation through repeated visits to traditional healers’ workspaces and the community, as well as informal conversations with community members. Following each interview and site visit, detailed notes were made.

### Study participants

Participants were recruited from the cutaneous leishmaniasis endemic communities in Kalu served by Ketetiya and Ardibo health centers [10]. Six adults, who were treated for cutaneous leishmaniasis and four with active lesions, five traditional healers, and three opinion leaders participated in the study. The number of participants was determined by the principle of saturation in the respective categories, where no new evidence was found with the engagement of any additional person.

### Eligibility criteria

Individuals who have been permanent residents of the study area for at least six months, willing to provide informed consent, and have voluntarily agreed to participate in the research were included in the data collection. On the other hand, those under the age of 18 years and those with cognitive impairments during data collection were excluded.

### Recruitment of participants

Health extension workers who reside in the community and are familiar with members of the community helped in identifying cutaneous leishmaniasis affected individuals as well as locally recognized influential leaders. Ten participants were selected using maximum variation to allow diversity of participants based on age, residence area, sex and those who are cured and with active lesions. Three local opinion leaders were selected through criterion sampling based on their age, experience, social influence, and strong acceptance within the community.

Traditional healers were recruited using the snowballing technique, i.e., referral by cutaneous leishmaniasis affected individuals and recommendations from other healers. Five participated in the interviews, while three allowed the lead author (MTE) to also observe the healing process.

### Trustworthiness

Lincoln and Guba's criteria (credibility, transferability, dependability, and confirmability) were used to ensure trustworthiness of the study findings [22]. The researcher's extended presence in the field, data collection method triangulation, cross-checking findings from interviews and observations, and peer debriefing strengthened credibility. Data saturation through repetition and triangulation using multiple data sources ensured dependability. Furthermore, detailed descriptions of the research methods and rich participant narratives enhanced transferability. The use of an audit trail documented the research process for confirmability.

### Medicinal plant identification

Plants used for the treatment of cutaneous leishmaniasis were identified through interviews with traditional healers and direct field observations. Detailed information was recorded, including local plant names, the specific parts utilized (e.g., leaves, bark), and preparation methods. Samples were recorded using photographs and detailed field notes. Botanical identification was conducted by comparing the samples with data from a prior ethnobotanical study carried out in Tehuledere District, a neighboring area to Kalu [23].

### Data processing and analysis

The interview recordings were transcribed verbatim and translated into English. The translated data and observation notes were imported into MAXQDA 2022 to facilitate coding. We used an inductive thematic analysis approach. The data was coded by the lead author (MTE) which was checked and confirmed by a senior researcher (MK). Identified codes were categorized based on their similarities, from which broader themes and subthemes emerged. Relevant quotations of the study participants’ expressions were used in presenting the study findings. Traditional healers were assigned pseudonyms.

## Findings

### Participants’ socio-demographic characteristics

A total of 18 individuals participated in the study, comprising 10 cutaneous leishmaniasis affected individuals, 5 traditional healers, and 3 local opinion leaders. The majority of participants were male (*n* = 11, 61%), with 14 (78%) of individuals aged between 20 and 49 years old. Educational status varied, ranging from no formal education to completion of secondary school, with a small number having attended tertiary education. Most participants were farmers (Table 1).

**Table 1:** Socio-demographic characteristics of participants (*n* = 18)

Characteristics of participants	Cutaneous leishmaniasis affected individuals (*n* = 10)	Traditional healers (*n* = 5)	Local opinion leaders (*n* = 3)
Sex			
Female	6	1	
Male	4	4	3
Age in years			
20–29	6	1	
30–49	3	3	1
Over 50	1	1	2
Occupation			
Farmer*	5	3	2
Government employee	1		
Small business owner*	1	2	1
Traditional healer*		5	
Currently not working	3		
Education level			
Tertiary education	2	1	
Secondary school	2	3	
Primary school	6		2
Never attended school		1	1
Marital status			
Single	5	1	
Married	4	4	3
Widow	1		
Religion			
Muslim	7	4	2
Orthodox Christian	3	1	1

*There is an overlap of occupations with traditional medicine practice. Two traditional healers were farmers, and another two owned small businesses as their main source of income

### Treatment decisions for cutaneous leishmaniasis

Interviews with cutaneous leishmaniasis affected people and healers revealed that individuals affected by cutaneous leishmaniasis follow patterns of care that typically progress from pursuing home-based treatment to visiting traditional healers, with only a few seeking allopathic healthcare. At the household level, cutaneous leishmaniasis is recognized based on its symptoms, and home-based remedies are sometimes used.

The lesion was on my nose. It was not painful, but it itched and gradually got bigger. I decided to try a home remedy and applied honey every morning (23-year-old male affected by cutaneous leishmaniasis).

A health professional affected by cutaneous leishmaniasis visited Boru Meda General Hospital for management of the disease. However, he reverted to home remedy because there was no treatment available at Boru Meda General Hospital at that time.

I went to Boru [Meda General] Hospital, but they had no treatment but confirmed that it was ‘Kunchir’ [cutaneous leishmaniasis]. Then I came home and applied the Tazma honey. Now, I don’t have any scars or marks (32-year-old male affected by cutaneous leishmaniasis).

If the problem persists, cutaneous leishmaniasis affected individuals visit traditional healers who may use herbal or spiritual treatments. Cutaneous leishmaniasis affected individuals sought traditional treatments based on recommendations from their family members, neighbors, and community members. One participant reported,

A person who had been healed… said ‘I did not see what kind of leaf the medicine was, but I am healed, so go there [to a traditional healer]…’. Then I went; that traditional treatment healed me (40-year-old female affected by cutaneous leishmaniasis).

And another.

…I found out about a traditional healer…. and he gave me the medicine that cured me. (70-year-old female affected by cutaneous leishmaniasis)

An opinion leader described the lack of awareness of the availability of allopathic treatment as key to explaining people’s use of traditional medicine:

They (cutaneous leishmaniasis affected individuals) don’t know that there is modern medicine that can heal cutaneous leishmaniasis; it is a problem of awareness. (Local opinion leader)

Some individuals affected by cutaneous leishmaniasis initially opted for household remedies because the disease is common in the community and often perceived as a minor condition.

I didn’t go to the health center because I believed that medical treatment wouldn’t cure me. I also didn’t think modern medicine was available for ‘Kunchir’. In our village, we consider it [cutaneous leishmaniasis] a minor condition, so I didn’t seek treatment there [health center]. No one advised me to go, so I applied a remedy [sap of Euphorbia abyssinica] myself, and it just got better. (23-year-old male affected by cutaneous leishmaniasis)

Limited access to allopathic healthcare facilities was mentioned as a barrier, leading individuals to prefer traditional healers within their communities. Additionally, the seasonal nature of agricultural work restricts income generation to specific times of the year, limiting access to costly allopathic treatments. The prolonged duration of allopathic treatment, which may require repeated trips to the hospital or even 28 days of hospitalization, poses a financial and time challenge for affected individuals and their families.

There is a treatment in Boru [Meda General] Hospital. I have heard that it will take up to a month, and you need to be admitted to get the treatment. Also, I need money when I stay there for a whole month, so I am thinking of going next year after I save the money I need for that. (28-year-old male affected by cutaneous leishmaniasis)

On the other hand, a small amount of money ranging from 50 to 100 ETB (0.38–0.77 USD) or something in-kind, such as sugar, khat (*Catha edulis*), juice, and incense, are given to the traditional healers for the medication to be effective and not as payment for the treatment, which is known as *Erensa*. In addition, they would bring *Hadiya* (a gift) to the traditional healer when they are healed.

Some cutaneous leishmaniasis affected individuals argued that people’s belief and confidence that traditional healers have specialized skills for treating certain ailments, including cutaneous leishmaniasis, was why some preferred seeking care from traditional healers. This trust is built either on personal experience or on accounts from others who have successfully recovered using traditional treatments. People may spend a large amount of money travelling to a traditional healer, and sometimes they bring expensive gifts like goats and camels.

Their [traditional healers’] treatment is not preferred because it’s free, but because of the outcome. It doesn't matter if there's a payment or not; it's because we see its effectiveness. We don't think that it's free because we are taking a lot of things with us willingly. (24-year-old female affected by cutaneous leishmaniasis)

Sociocultural influences also played a pivotal role in shaping healthcare-seeking behaviors. Certain diseases, including cutaneous leishmaniasis, were believed by some to be treatable only through traditional methods. A traditional healer explained,

They [cutaneous leishmaniasis affected individuals] choose traditional medicine because there is a community viewpoint that it [cutaneous leishmaniasis] is cured by traditional treatment. They believe that …‘kunchir’[cutaneous leishmaniasis] can be cured only by traditional medicine, and not by modern treatment. (Traditional Healer-SH)

The allopathic treatment option was often considered the last option, only when traditional options had been exhausted.

…but now we are ready to seek the modern option. We have seen that the traditional medicine has not healed us; that is all I'm saying. (25-year-old male affected by cutaneous leishmaniasis)

### Sources of traditional treatment for cutaneous leishmaniasis

Traditional treatments used for cutaneous leishmaniasis include home remedies and treatments from traditional healers.

### Home remedies

This level of care is based on locally shared understandings of the symptoms and experiences in self-management.

- **Honey**

Honey is usually applied to the wound for its medicinal properties as a first line of treatment in their household. An account by a cutaneous leishmaniasis affected individual;

I smeared the lesion with honey, and this helped the wound not to have a foul odor…. I put the honey on the wound at night and cleaned it with soap and water in the morning. …. The wound was healed after I used the honey for some time. (32-year-old male affected by cutaneous leishmaniasis)

(b) **Use of dried bat meat**

In Kalu, a widely held belief is that bat urine is a cause of cutaneous leishmaniasis (10). Some people believe that applying dried and powdered bat meat can be used as a remedy for cutaneous leishmaniasis.

I killed the bat and dried the meat, then crushed and applied it over the affected area, and the wound has healed (28-year-old male affected by cutaneous leishmaniasis).

(c) **Cactus (Euphorbia abyssinica)**

The application of the sap of *Euphorbia abyssinica* to cutaneous leishmaniasis lesions is a common practice reported by community members. Cutaneous leishmaniasis affected individuals stated that while this substance can cause inflammation of the skin and surrounding areas, it is believed to eventually lead to healing of the lesion.

When I found out that the lesion was kunchir, I applied the latex from a plant called Qolqolo [Euphorbia abyssinica]. When you smear it on the lesion, it pulls up not only the wound but also part of the surrounding skin. It bores inside and uproots the lesion with the skin. (23-year-old male affected by cutaneous leishmaniasis)

(d) **Heated metal objects**

The application of heated metal objects such as knives, nails, sickles, or other metallic tools to cutaneous leishmaniasis lesions is reported to assist in healing through cauterization. Individuals affected by cutaneous leishmaniasis may perform this procedure themselves or be assisted by others. The duration and frequency of the application vary among individuals, depending on how quickly their wounds heal. A traditional healer shared his personal experience of using a heated knife to cauterize his cutaneous leishmaniasis lesion, stating:

I heated a knife and applied it to the wound. I applied it myself … it made the edges pull inward and dried the wound. (Traditional Healer-ME)

### Traditional healers’ settings and practice

Observations of traditional healers’ practices revealed a variety of settings and healing approaches. Three of the five traditional healers were observed (pseudonyms: ME, AJ, and TA). Consultations with six people for skin-related conditions were observed; none of these individuals were treated for cutaneous leishmaniasis.

The traditional healers predominantly utilized plant-based remedies. TA incorporated spiritual healing into the plant-based remedies he provides, while others provided plant-based remedies only.

ME’s practice setting was organized similarly to a modern clinic in Kalu. ME is licensed to practice traditional medicine. The waiting area contains a television, a couch, and a bench outside for overflow. Various traditional medicines prepared by combining different plants and minerals, used for different diseases, are displayed on a shelf in labeled containers.

Four consultations were observed. Clients are seen individually, which ensures confidentiality. During the consultations, ME asked questions to explore symptoms and, when deemed necessary, examined the affected area. After listening to the client’s concerns attentively, he recommended treatment based on the diagnosis, selecting from his range of pre-prepared remedies. These were dispensed directly to the client with verbal instructions on use. One client had a respiratory complaint that ME diagnosed as asthma. The other three clients had various skin conditions, which he diagnosed as *Chifie* (eczema), *Qoreqor* (tinea capitis), and *Shifta* (“skin rash”). There was no spiritual component observed during his consultations.

AJ’s healing space is located in a small room (about 3 × 3 m) adjacent to her home. A mattress is placed on the floor, and people, both clients and their caregivers, gather around, chatting and sharing stories. Consultations occur in the room, which limits the level of privacy and confidentiality. Two consultations on skin-related conditions, which AJ diagnosed as *Chifie* (eczema) and *Mich* (cold sore), were observed. AJ greeted clients warmly on arrival, inquired about their condition, and examined the affected skin. AJ prepared the treatment by grinding the leaves of different plants together in a mortar until they formed a paste. She also provided extra herbs for clients to take home for later use after instructing them on how to use the medicine.

TA has multiple houses, within a large compound, where clients who travel long distances for a consultation or those who stay for prolonged periods, including those with mental illness, can be accommodated. TA practices traditional healing in one of the houses, where people gather for *Dua* (prayer). He has *Khadam* (servants) who assist him, and some clients choose to share their problems with the servants when they feel uneasy speaking directly to him. Consultations take place in a shared space separated by a curtain, offering only a minimum of privacy but maintaining an informal atmosphere. Two consultations were observed: one involved a client seeking treatment for a skin condition, which TA diagnosed as *Quaqucha (pityriasis versicolor)*, while the other sought a blessing for an upcoming journey. After listening to the client’s complaint, TA prepared a medicine using various leaves of different plants by crushing them together and heating them on a charcoal stove while reciting Quranic verses, believed to enhance the medicine’s effectiveness. His approach combined herbal and spiritual elements. TA is widely regarded by the community as possessing supernatural healing and problem-solving powers.

### Traditional healers’ interventions

Interviews with traditional healers revealed that they utilize traditional medicinal knowledge passed down through generations, often from parents or close relatives, to provide therapeutic interventions. This level of care encompasses the use of plants and spiritual healing practices to manage cutaneous leishmaniasis and promote healing.

- **Plant sources**

Traditional healers mainly used plant-based traditional medicines for the management of cutaneous leishmaniasis. The management of cutaneous leishmaniasis using plant-based remedies utilizes various plants and different parts of the plants, such as leaves and bark, used alone or in combination. Plants are prepared for topical use by heating, grinding, and crushing (Table 2).

**Table 2:** Medicinal plants used for treatment of cutaneous leishmaniasis in the study area

Local name	Scientific name	Parts used	Preparation/application
Yebere chew	*Oxalis corniculata L*	Leaves	Crush and warm the leaves and tie them on the lesion
*Hulegeb*	*Salvia nilotica*	Leaves	Crush the leaves with *Ocimum lamifolium* leaves then tie them on the lesion
*Boter*	*Plectranthus sp.*	Leaves	Cut and rub the leaves on the lesion Crush the leaves with *Hulegeb* and *Ocimum lamifolium* leaves and apply the paste
Nech shinkurt	*Allium sativum* L.	Bulb	Heat and directly apply the bulb to the lesion Crush the bulb with black cumin and cobwebs, then apply the paste
Qolqolo	*Euphorbia abyssinica*	Sap	Apply the sap directly on the lesion
Bisana	*Croton macrostachyus*	Bark	Dry and grind the bark, then mix the powder with sulfur, petroleum jelly, Sibir, Qurqura powder, and alum stone to make a paste
Qurqura	*Ziziphusmauritiana*	Leaves	Dry and grind the leaves, then mix the powder with sulfur, petroleum jelly, Sibir, Bisana bark powder, and alum stone to make a paste

Community members have limited knowledge of the medicinal plants employed, which traditional healers collect in secret to maintain the exclusivity of their knowledge.

**Fig. 1:**
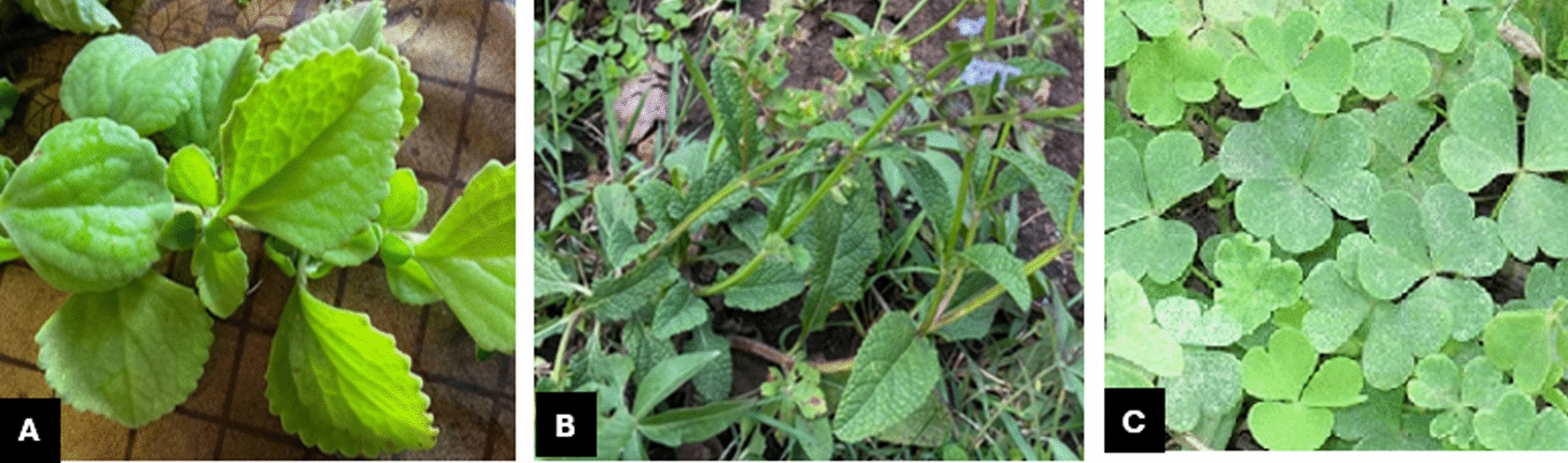
Photo of plants used for treatment of *kunchir* (cutaneous leishmaniasis) (Amharic name/botanical name). **A** Boter*/Plectranthus sp.* **B** Hulegeb/*Salvia nilotica.* **C** Yebere chew/ *Oxalis corniculata L.*

The most commonly used part of plants in treatments for cutaneous leishmaniasis is the leaves. Traditional healers mentioned different leaves: *Yebere chew (Oxalis corniculata L.), Hulegeb (Salvia nilotica), and Boter (Plectranthus sp.)* (Fig. 1). AJ shared her experience of using *Boter* combined with *Hulegeb* and *Damakesie* (*Ocimum lamifolium)* on the lesion.

I combine leaves of Boter, Hulegeb, and Damakesie. I crush them together and apply the paste to the lesion. The paste mustn't be warm. (Traditional Healer-AJ)

Garlic (*Allium sativum* *L.*) is considered an effective traditional remedy for the treatment of cutaneous leishmaniasis. Traditional healers recommend using heated garlic cloves to cauterize the lesion or crushing fresh garlic, mixing it with black cumin and cobwebs, then applying it as a paste. One former cutaneous leishmaniasis patient explained her experience as:

He (the traditional healer) told me to combine cobwebs, black cumin, and garlic and apply the paste to the lesion. First, the black cumin is crushed along with the garlic and then the cobweb is added and warmed up on a fire. After it is warm, it is applied once a day, either in the morning or in the evening. When I applied it, it healed the lesion immediately. (24-year-old female affected by cutaneous leishmaniasis)

**Fig. 2:**
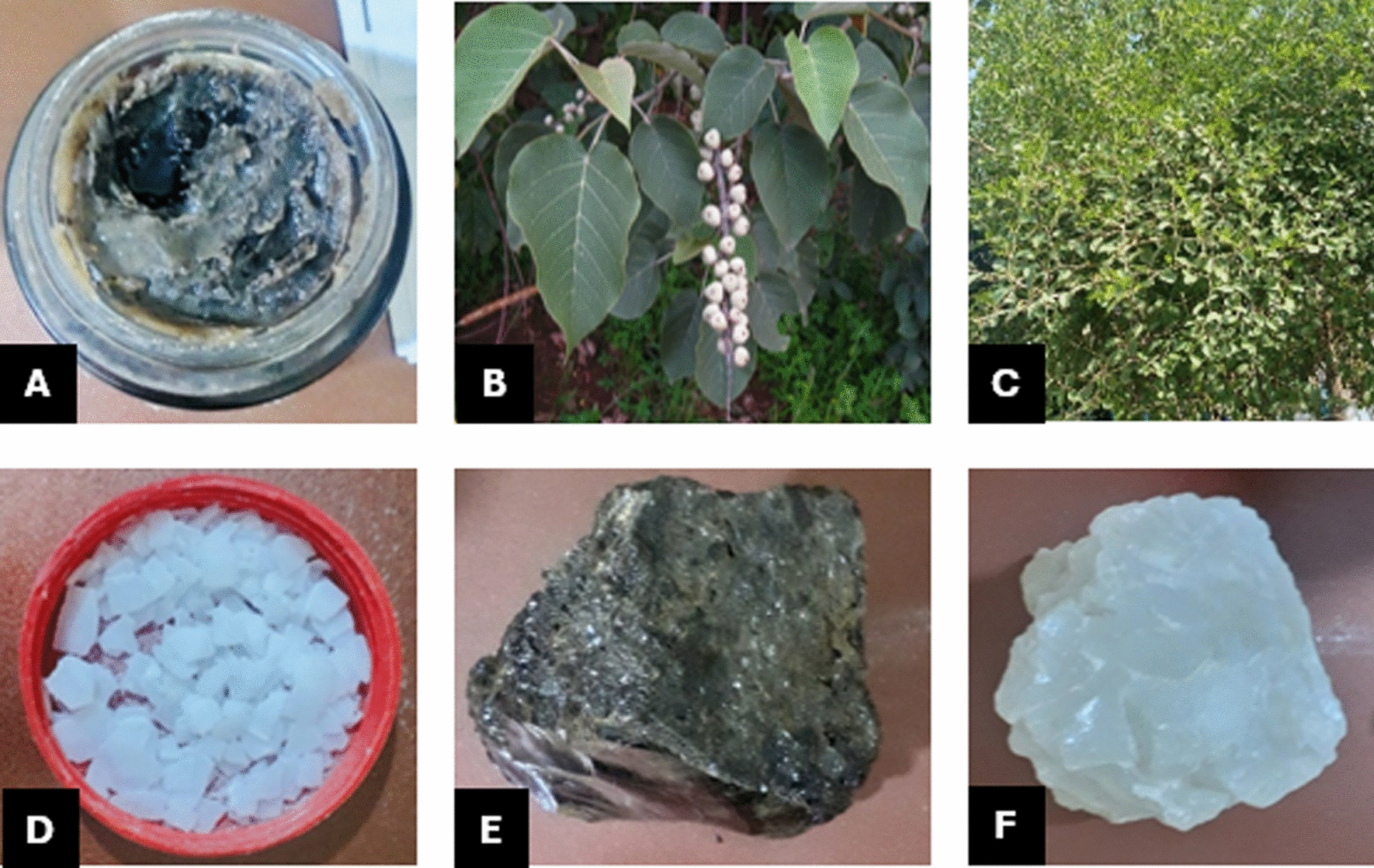
Images of plants and minerals used to prepare *Ketran* for the treatment of cutaneous leishmaniasis. **A** *Ketran* (the final paste). **B** Bisana*/Croton macrostachyus.* **C** Qurqura/*Ziziphus mauritiana.* **D** Sulfur. **E** Sibir (dried aloe Vera). **F** Sheb dingay (Alum stone)

ME prepares a paste for cutaneous leishmaniasis, which he calls *Ketran*, by combining a tablespoon of sulfur, petroleum jelly, *Sibir* (dried aloe vera), dried and crushed *Bisana* (*Croton macrostachyus)* bark and *Qurqura* (*Ziziphus mauritiana*) leaves, and crushed *Sheb dingay (Alum stone*) (Fig. 2). *Croton* *macrostachyus* bark and *Ziziphus mauritiana* leaves are dried in shade because ME stated they lose their medicinal properties if exposed to direct sunlight.

(b) **Spiritual healing**

In the study area, the main religious groups are Muslims and Orthodox Christians. Despite their different beliefs, people sometimes use spiritual healing practices from other faiths; Muslims may use holy water from a church. *Silet* involves making an oath to bring a gift as a reward to the church or the traditional healer when one gets cured. *Tufta* is a treatment ritual which involves a traditional healer or religious leader chewing khat leaves (*Catha edulis*) and spitting on the lesion or into a bottle of water for the cutaneous leishmaniasis affected individuals to drink. The community has accepted it as an effective means of treatment for any health problem. Cutaneous leishmaniasis affected individuals described healers who perform *Tufta* as having supernatural healing power passed on from generation to generation.

The traditional healer first spat the chewed khat here [on her lip with the lesion], then he gave me the second one to take home and smear. People here believe that his “Tufta” is a medicine. (38-year-old female affected by cutaneous leishmaniasis)

Those who use Orthodox healing resources used water blessed by a priest as holy (*Tsebel*) to help treat cutaneous leishmaniasis. Participants indicated applying *Emnet* (ash from the incense burned during Orthodox mass service and/or soil from the church compound) mixed with holy water and applied to the cutaneous leishmaniasis lesion. An individual whose cutaneous leishmaniasis had healed shared his experience as follows:

I brought the Emnet from Teklehaymanot church and applied it to the lesion by mixing it with holy water every morning. After I applied that every day for about five days, the wound disappeared (32-year-old male affected by cutaneous leishmaniasis).

### Lifestyle advice given to affected individuals

In addition to treatments for cutaneous leishmaniasis, traditional healers gave lifestyle advice aimed at promoting healing. Advice included sexual abstinence, refraining from routine economic activities, and social gatherings, as well as dietary restrictions.

*Tibiq* (sexual abstinence) is widely believed to promote healing or prevent exacerbation of cutaneous leishmaniasis lesions.

If the person has ‘Kunchir’, he/she should be Tibiq. It means that he/she shouldn’t have sexual intercourse so that the lesion does not worsen. (25-year-old male affected by CL)

Avoiding chores like cooking, baking, and farm work was said to prevent sweat, which worsens cutaneous leishmaniasis wounds by keeping them moist. They also believed touching soil (apart from holy soil from church grounds) aggravates cutaneous leishmaniasis, so affected individuals are advised against farming.

Traditional healers differed in advocating *Tila*, the avoidance of social interaction, whilst using traditional medicine. Tila is practiced so that the affected individual avoids the shadow of others, which is believed to reduce the effectiveness of treatment and aggravate the lesion.

… another person's shadow casts on him/her [person with cutaneous leishmaniasis], and the medicine that has been applied does not work. (Traditional Healer-TA)

The belief in the effect of *Tila* was strongly held. A traditional healer, who advised against self-exclusion, reported that individuals ignored this recommendation.

Even when I reassure them that it [Tila] is not an issue, some refuse to listen. (Traditional Healer-ME)

Dietary recommendations included the avoidance of protein-rich animal products such as eggs, meat, and milk, and alcoholic beverages, which are believed to delay healing.

We prohibit individuals taking these medications from consuming eggs, milk, and beef because these foods have high protein, and alcohol consumption because they will slow healing. (Traditional Healer-SH)

## Discussion

We explored the practice of traditional treatment for cutaneous leishmaniasis in Kalu, Ethiopia, revealing cutaneous leishmaniasis affected individuals use locally available traditional medicine as their first choice. Other studies conducted in Ethiopia, Bolivia, and Peru also show that many participants first try traditional treatment options before they visit allopathic healthcare facilities [9, 16, 17, 20]. Most participants stated that allopathic healthcare was the last resort for cutaneous leishmaniasis care when other, traditional treatment options failed, and/or the condition worsened. This finding is in line with other studies that reported a higher preference for traditional treatment for the management of cutaneous leishmaniasis only resorting to allopathic treatments when traditional medicines fail [16, 17]. This indicates a strong cultural inclination towards traditional healing practices within communities with other factors such as limited access to health facilities offering cutaneous leishmaniasis treatment, lack of awareness about available allopathic options, the perceived high cost and long duration of allopathic treatment, trust in traditional healers’ abilities and the perceived effectiveness of traditional medicines, as well as advice and recommendations from trusted members of the social network influencing the decision [16, 21, 24].

The commonly used traditional medicine for cutaneous leishmaniasis in the study area included plants, animals, spiritual practices, and heated metallic objects. Participants mentioned the primary use of plant sources, with leaves being the most frequently utilized plant parts [8, 17, 25]. Many medicinal plants contain bioactive compounds with demonstrated antimicrobial, anti-inflammatory, and antiparasitic properties [26]. *Euphorbia abyssinica*, frequently cited by participants, has been shown to have effective antileishmanial properties in preclinical studies [27]. Similarly, garlic (*Allium sativum*) exhibits potent antimicrobial and antiparasitic effects, primarily attributed to its active compound, allicin, which aligns with findings that garlic enhances immune responses and directly inhibits *Leishmania* parasites in vitro and animal models [28]. In addition, studies on the therapeutic application of *Oxalis corniculata L.* and *Salvia nilotica* have revealed anti-inflammatory and wound-healing properties, which may help explain the community’s belief in its effectiveness for treating cutaneous leishmaniasis [29, 30]. However, clinical evidence in humans remains limited, highlighting the need for further research to standardize preparations and dosing.

The practice of applying heated metallic objects to cutaneous leishmaniasis lesions aligns with the principles of thermotherapy, a recognized, safe, and cost-effective treatment for localized cutaneous leishmaniasis, especially in settings with limited resources. While traditional techniques lack the precision and safety of modern medical equipment, they represent a form of thermotherapy rooted in experiential knowledge, highlighting a community-level recognition of heat’s therapeutic potential [11, 25, 31]. Spiritual healing practices also played a significant role in the traditional management of cutaneous leishmaniasis in the study community. These included rituals, the use of holy water, and the application of Emnet. However, while such practices may offer psychological comfort and social support, they can also contribute to delays in diagnosis and treatment [8].

The interactions observed between traditional healers and their clients were characterized by respect, patience, and the preservation of dignity. Consultations were generally calm and welcoming, providing ample time for clients to share their concerns within a culturally familiar environment. However, limitations such as a lack of privacy in some settings and reliance on anecdotal diagnostic methods may affect the quality and confidentiality of care.

In Kalu, individuals with cutaneous leishmaniasis are advised to avoid certain animal-source proteins, abstain from sexual activities, and limit social interaction, practices believed to exacerbate lesions. Interestingly, while participants often viewed allopathic treatment as a cause of lost income due to travel and extended hospital stays, they did not recognize that traditional healing practices also impose significant economic burdens. Restrictions on routine agricultural work and household chores may deprive families of vital income in this agrarian setting, negatively affecting both household welfare and broader livelihood outcomes. Furthermore, celibacy and social exclusion, though culturally significant, can have serious impacts on mental wellbeing by fostering loneliness, depression, and social isolation [9, 10].

## Strengths and limitations

Triangulation of evidence by methods of different data collection and sources allowed for the exploration of a wide range of ideas related to the use of traditional medicine for cutaneous leishmaniasis. It has further helped to ensure data saturation to explain the state of traditional treatment for cutaneous leishmaniasis in the particular study community. Yet, despite best efforts to ensure rigor, one may not rule out potential social desirability and recall bias on the one hand, and the small number of participants. Recall bias may have affected the accuracy of participants accounts, particularly among former patients, as their memories of their treatment experiences may have been altered over time. Additionally, small number of traditional healers observed and no direct observation of cutaneous leishmaniasis treatment might have limited insight into healing practices for management of cutaneous leishmaniasis.

## Conclusions

This study highlights the role of traditional medicine in the management of cutaneous leishmaniasis in Kalu, Ethiopia. Treatment decisions are influenced by structural barriers such as limited access to allopathic healthcare, financial constraints, and sociocultural beliefs. The findings underscore the need for strengthening health education to raise awareness about allopathic treatment options and decentralizing treatment to facilitate access.

Collaborative engagement between health researchers and traditional healers may provide evidence for improving early access to allopathic care. Future research should focus on evaluating the safety and efficacy of traditional medicines to identify potentially novel phytomedicines and guide evidence-based policy decisions for cutaneous leishmaniasis.

## Recommendations

Based on the findings from this study, it would be useful to carry out further research to evaluate the safety and effectiveness of traditional healing practices for cutaneous leishmaniasis in particular and skin diseases in general. In addition, the government is called on to pay attention to the role of traditional healers to find ways of engaging them for positive outcomes of interventions against skin diseases.

## Data Availability

The datasets used and/or analyzed during the current study are available from the corresponding author on reasonable request.
